# Left-Sided Diaphragmatic Hernia Following Pediatric Liver Transplantation: First Reported Case From the Middle East

**DOI:** 10.7759/cureus.96933

**Published:** 2025-11-15

**Authors:** Dana Sarmini, Mohammed O Ibrahim, Farheen Khan, Mamoun AlMarzouqi, Sobhy Kotb

**Affiliations:** 1 Pediatrics, Al Jalila Children's Speciality Hospital, Dubai, ARE; 2 College of Medicine, Mohammed Bin Rashid University of Medicine and Health Sciences, Dubai, ARE; 3 Pediatric Surgery, Al Jalila Children's Speciality Hospital, Dubai, ARE

**Keywords:** acquired diaphragmatic hernia, autoimmune hepatitis type 2, case report, gastric herniation, immunosuppression, left diaphragmatic hernia, liver-kidney microsomal antibody (lkm-1), paediatric liver transplantation, pediatrics

## Abstract

Acquired diaphragmatic hernia (DH) is an uncommon but clinically important complication after pediatric liver transplantation (LT), most often affecting the right hemidiaphragm. Left-sided defects are exceptionally rare in children, with only isolated cases reported. To the best of our knowledge, this is the first documented pediatric case of left-sided DH after LT from an Arab Middle Eastern country. A 16-year-old female with autoimmune hepatitis type 2 (AIH-2) on long-term immunosuppression presented two years post-transplant with acute epigastric and left upper quadrant pain that progressed to chest discomfort and dyspnea. Initial radiography suggested possible eventration, while subsequent advanced imaging revealed a left diaphragmatic defect with herniation of the gastric fundus. Surgical exploration confirmed gastric and omental herniation with ischemic changes, requiring conversion from thoracoscopy to open repair and primary closure. The patient recovered well without recurrence. This case underscores the diagnostic difficulty of post-transplant DH, particularly when delayed and left-sided, and highlights the importance of maintaining a high index of suspicion in LT recipients with unexplained abdominal or respiratory symptoms. Its atypical laterality, gastric involvement, and regional novelty broaden current understanding of this rare complication and emphasize the need for timely imaging and intervention.

## Introduction

A diaphragmatic hernia (DH) occurs when abdominal organs move into the thoracic cavity through a defect in the diaphragm. Such defects may be present at birth or develop later in life [1]. Although most acquired DHs follow blunt or penetrating trauma, they are now being recognized as rare complications of upper abdominal surgery, including hepatectomy and orthotopic liver transplantation (LT) [2]. Among pediatric LT recipients, DH is an uncommon but serious complication that can be life-threatening. Presentations vary and may include abdominal pain, respiratory distress, bowel obstruction, or strangulation, requiring urgent surgical repair [3].

The exact incidence of pediatric left-sided DH following LT is exceptionally low, with only isolated cases reported in the literature and no large cohort able to provide a precise percentage [3]. Most pediatric series and systematic reviews report a strong right-sided predominance of post-transplant DH, with right-sided defects accounting for the overwhelming majority of cases, while left-sided hernias remain exceedingly uncommon and often appear as only a single case within multi-year institutional cohorts. [4]. In one review that included 41 pediatric patients, only one left-sided hernia was identified [4]. By contrast, across all pediatric LT recipients, the overall incidence of post-transplant DH is estimated at approximately 0.8-2.9% [5,6].

The timing of presentation is also variable. The typical timing of presentation is within the first year post-transplant, with a median interval of 2-9 months; however, late presentations up to 10 years have also been reported [7]. Most symptomatic cases tend to appear within the early months after transplantation. However, a subset of patients may show no overt symptoms, with the diagnosis made only when imaging is performed for another reason [7]. If the condition is missed or its recognition is delayed, the likelihood of complications increases markedly, and the hernia may evolve into bowel obstruction, perforation, volvulus, pleural effusion, or respiratory compromise [8]. Between 20% and 27% of pediatric patients ultimately require bowel resection because of strangulation or necrosis, and fatalities have been described in cases where the diagnosis was missed or made too late [9,10]. When the diagnosis is made early and the defect is repaired promptly, the prognosis is usually excellent. Recurrence after surgery is rare, with most published series noting rates below 5% at five-year follow-up [10].

In adults, post-transplant DH occurs infrequently, usually reported in fewer than 1% of cases, and tends to involve the left side more often than in children [4]. In pediatric recipients, the complication occurs more often, estimated in about 1-3% of liver transplants, and it tends to involve the right hemidiaphragm. When diagnosis is delayed, these patients face a greater risk of significant morbidity and postoperative complications [4-6]. Diagnosis relies on a high index of suspicion in any post-LT patient with acute or subacute respiratory or obstructive gastrointestinal symptoms [11]. Even though chest radiography can suggest the diagnosis, computed tomography (CT) remains the preferred imaging modality for confirming a diaphragmatic defect and defining the extent of herniated abdominal contents and related complications [12]. Surgical repair, either through laparotomy or thoracoscopy, remains the definitive treatment, with early reduction and secure closure essential to prevent incarceration or recurrence [8,13].

Autoimmune hepatitis type 2 (AIH-2), characterized by anti-liver-kidney microsomal (anti-LKM) antibodies directed against cytochrome P450 2D6, represents one of the most aggressive pediatric autoimmune liver diseases and is a recognized indication for LT [14,15]. Patients often require extended triple immunosuppressive therapy, a treatment that can delay wound healing and increase the likelihood of post-transplant structural complications, including acquired DH [14]. 

To date, we have been unable to identify any published case reports of left-sided DH in pediatric patients after LT originating from an Arab country within the Middle East, including the Gulf Cooperation Council (GCC) region or the United Arab Emirates (UAE) [12]. The available literature describes acquired DH as a rare complication after pediatric LT, with most reported cases involving right-sided hernias and originating from centers in Turkey, Korea, China, Brazil, and other regions outside the Middle East and GCC [3,5,6,7]. Reports of left-sided DH are exceptionally limited, with only a small number of publications describing such cases, including the series by Çeltik et al. and a few isolated reports [7]. However, none of the published cases we reviewed appeared to originate from an Arab country in the Middle East, the GCC, or the UAE. Based on our literature search, we did not identify pediatric cases from this geographic region, indicating that the present report may represent the first documented occurrence in this context within the indexed medical literature.

In this context, we report a left-sided acquired DH following LT in a 16-year-old pediatric liver transplant recipient in the UAE, a laterality and age combination less frequently represented in the literature, thereby adding geographic and clinical depth to an uncommon but consequential post-transplant complication.

## Case presentation

A 16-year-old female with a history of AIH-2, positive for LKM antibodies, who had previously undergone cadaveric orthotopic LT in April 2022, presented with acute abdominal pain. Her post-transplant course was notable for biopsy-proven rejection and biliary stricture, for which she was maintained on triple immunosuppressive therapy (tacrolimus, everolimus, and mycophenolate). She also had a history of cytomegalovirus infection, iron deficiency anemia, and vitamin D deficiency. At the time of presentation, she remained adherent to her immunosuppressive and supportive medications.

In July 2024, she presented to the emergency department (ED) with a two-day history of severe epigastric and left upper quadrant abdominal pain associated with multiple episodes of non-bilious, non-bloody vomiting. These symptoms were preceded by pain and movement limitation in her left upper limb, which was subsequently evaluated by the orthopedic team and deemed unrelated to her thoraco-abdominal pathology. On arrival, she was tachycardic and hypertensive but afebrile, pale, and in visible distress. The abdominal examination showed localized epigastric tenderness, without guarding or rebound, and the surgical transplant scar appeared well-healed. The left arm showed tenderness with restricted active movement, while the passive range of motion remained intact. Neurological function was preserved without any focal deficits detected. 

**Figure 1:**
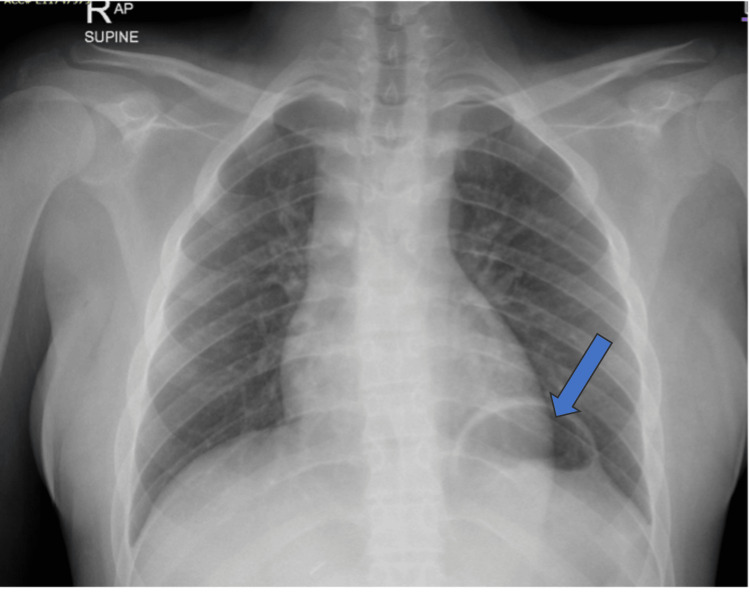
Chest radiograph on admission demonstrating clear lung fields but focal bulging beneath the left hemidiaphragm (arrow), initially interpreted as possible eventration.

Laboratory investigations on admission demonstrated normocytic anemia (Hb 11.1 g/dL) and mild elevation in liver enzymes (aspartate aminotransferase (AST) 117 U/L, alanine aminotransferase (ALT) 90 U/L, alkaline phosphatase (ALP) 262 U/L, and gamma-glutamyl transferase (GGT) 136 U/L), while renal function and electrolytes were within normal limits. Inflammatory markers were initially normal as well (C-reactive protein (CRP) 1.2 mg/L). Initial chest radiography revealed a focal bulge beneath the left hemidiaphragm, reported as possible diaphragmatic eventration (Figure 1), with clear lung fields and no evidence of consolidation. This interpretation was based on the smooth elevation of the hemidiaphragm without definite visualization of herniated abdominal contents, which can mimic eventration in early imaging. Abdominal ultrasonography confirmed preserved graft vasculature and normal echotexture of the transplanted liver, with no ascites, abscess, or dilated bowel loops, although a trace of left pleural effusion was noted. 

**Figure 2:**
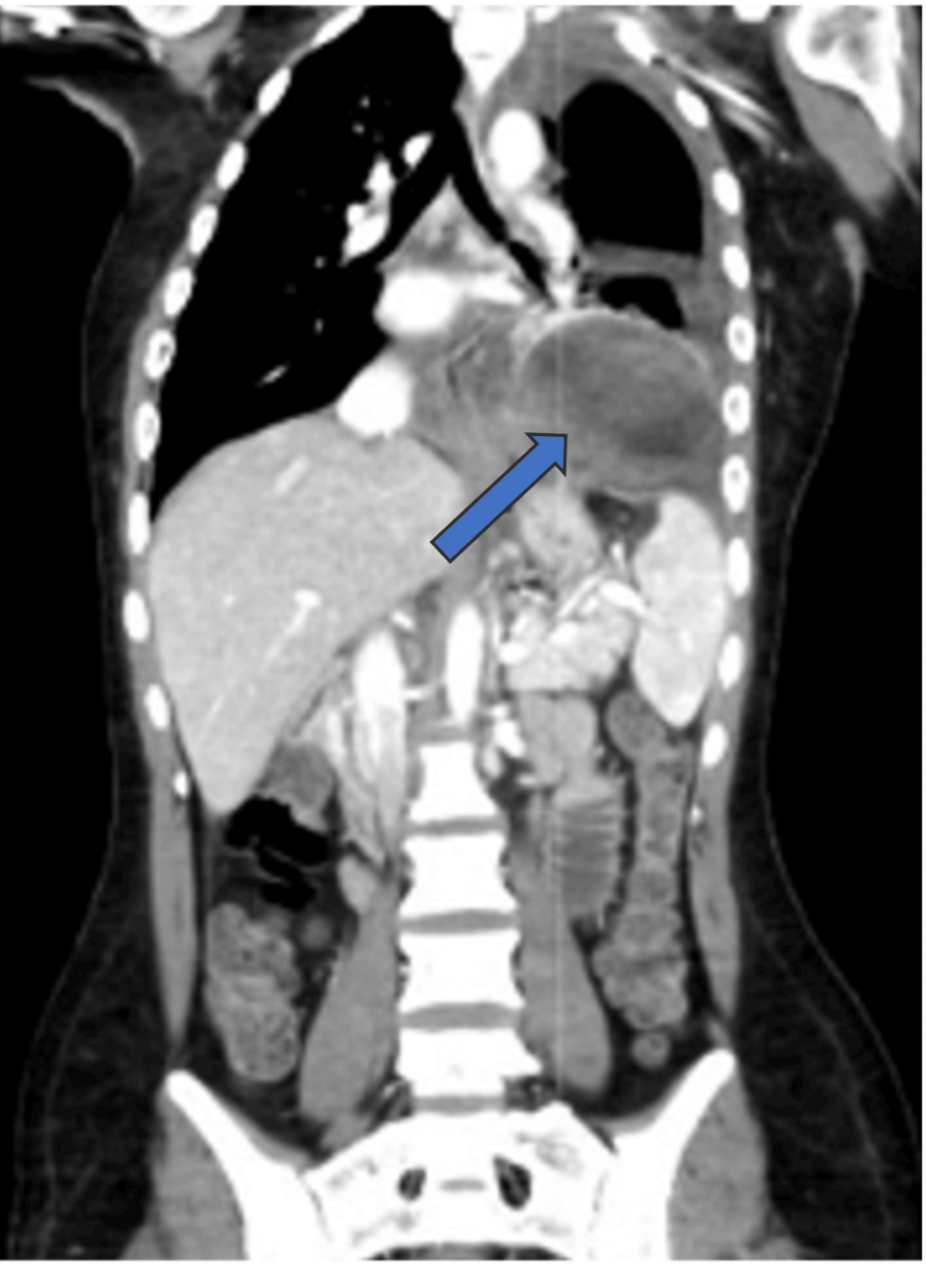
CT chest and abdomen (July 11, 2024) showing a 2.5 cm defect in the left hemidiaphragm (arrow) with herniation of the gastric fundus into the pleural cavity, associated with pleural effusion and atelectasis.

At this stage, the working differential diagnoses included infectious gastritis, given the acute abdominal pain, vomiting, and mild liver enzyme elevation in an immunosuppressed patient. Blood cultures were obtained, and empirical broad-spectrum antibiotics were initiated. Musculoskeletal pain was considered for the arm complaint, which was excluded as a primary diagnosis after orthopedic evaluation. Biliary complications such as stricture or cholangitis were also considered but excluded based on imaging and the absence of cholestasis or jaundice. Acute rejection was considered unlikely due to the stable appearance of the graft, and negative inflammatory markers reduced the probability of infectious causes. Pulmonary pathology, such as pneumonia and effusion, was considered; however, serial chest radiographs revealed only a persistent elevation of the left hemidiaphragm. Psychological causes of pain were also considered in view of all the negative initial workup, and it was planned for the mental health team to meet her. However, it was deferred when the diagnostic turning point occurred and the patient developed persistent, severe left-sided chest pain accompanied by a sharp rise in inflammatory markers despite being on empirical broad-spectrum antibiotic. A detailed summary of serial laboratory results throughout admission and follow-up is presented in Table 1. Immediate electrocardiogram (ECG) showed normal sinus rhythm and a repeat chest X-ray showed the same finding of the left diaphragmatic eventration, which prompted advanced imaging, including CT of the chest and abdomen, which demonstrated a 2.5 cm defect in the central left hemidiaphragm with herniation of the gastric fundus into the pleural cavity, associated with moderate pleural effusion and passive atelectasis of the left lower lobe (Figure 2). These findings established the diagnosis of an acquired left diaphragmatic hernia.

**Table 1:** Serial laboratory results during hospitalization and follow-up. WBC, white blood cell count; CRP, C-reactive protein; AST, aspartate aminotransferase; ALT, alanine aminotransferase; ALP, alkaline phosphatase; GGT, gamma-glutamyl transferase; INR, international normalized ratio; H, high; L, low

Parameter	Reference range	July 7, 2024 (Presentation)	July 11, 2024 (deterioration)	July 12, 2024 (Post-op)	August 12, 2024 (Follow-up)
Hemoglobin (g/dL)	12-15	11.1 (L)	10.2 (L)	8.9 (L)	9.5 (L)
WBC (×10³/µL)	3.6-11.0	6.6	18.2 (H)	12.4 (H)	8.6
Platelets (×10³/µL)	150-410	6.6	296	253	245
CRP (mg/L)	0-5	1.2	165.1 (H)	196.8 (H)	1.1
AST (U/L)	0-27	117 (H)	56 (H)	71 (H)	38 (H)
ALT (U/L)	0-23	90 (H)	45 (H)	46 (H)	30 (H)
ALP (U/L)	44-107	262 (H)	185 (H)	163 (H)	238 (H)
GGT (U/L)	0-29	136 (H)	-	-	188 (H)
Total bilirubin (mg/dL)	0-1.2	0.6	1.27 (H)	1.88 (H)	0.54
Albumin (g/dL)	3.2-4.5	4.2	3.5	2.7 (L)	3.8
INR	0.97-1.30	-	1.07	1.18	-
Creatinine (mg/dL)	0.59-0.86	0.69	0.55 (L)	0.64	0.97 (H)
Urea (mg/dL)	15.6-40.6	29	23	31	65 (H)
Sodium (mmol/L)	136-145	137	138	136	138
Potassium (mmol/L)	3.5-5.1	3.8	4.8	4.7	4.3
Procalcitonin (ng/mL)	0.03	-	-	0.31	0.03

The patient was taken to the operating theater (OT) for urgent repair. Initially, thoracoscopic access was attempted with a 5-mm camera port and two 3-mm working ports. Upon entry, the stomach displayed significant distension and a dusky appearance, with approximately 400 mL of hemorrhagic pleural fluid aspirated. Needle aspiration decompressed the stomach, which resulted in the extraction of approximately 200 mL of dark blood. The diaphragmatic defect was small but densely adherent to the stomach and omentum, which appeared ischemic. Due to difficulty reducing the herniated viscera, the procedure was converted to an open approach through a left subcostal incision extended to the midline below the xiphoid process. The intra-abdominal portion of the stomach appeared normal, but the fundus and omentum were incarcerated and adherent within the diaphragmatic defect. The defect was carefully enlarged to facilitate reduction, which proved technically challenging because of dense adhesions. The herniated fundus was dusky but partially improved after reduction, while the omentum demonstrated gangrenous changes and was resected. The diaphragmatic defect was repaired with interrupted non-absorbable sutures. The abdominal wall was closed in layers with absorbable sutures for the muscle and fascia, a monofilament absorbable suture for the skin, and reinforced with a topical cyanoacrylate adhesive. 

**Figure 3:**
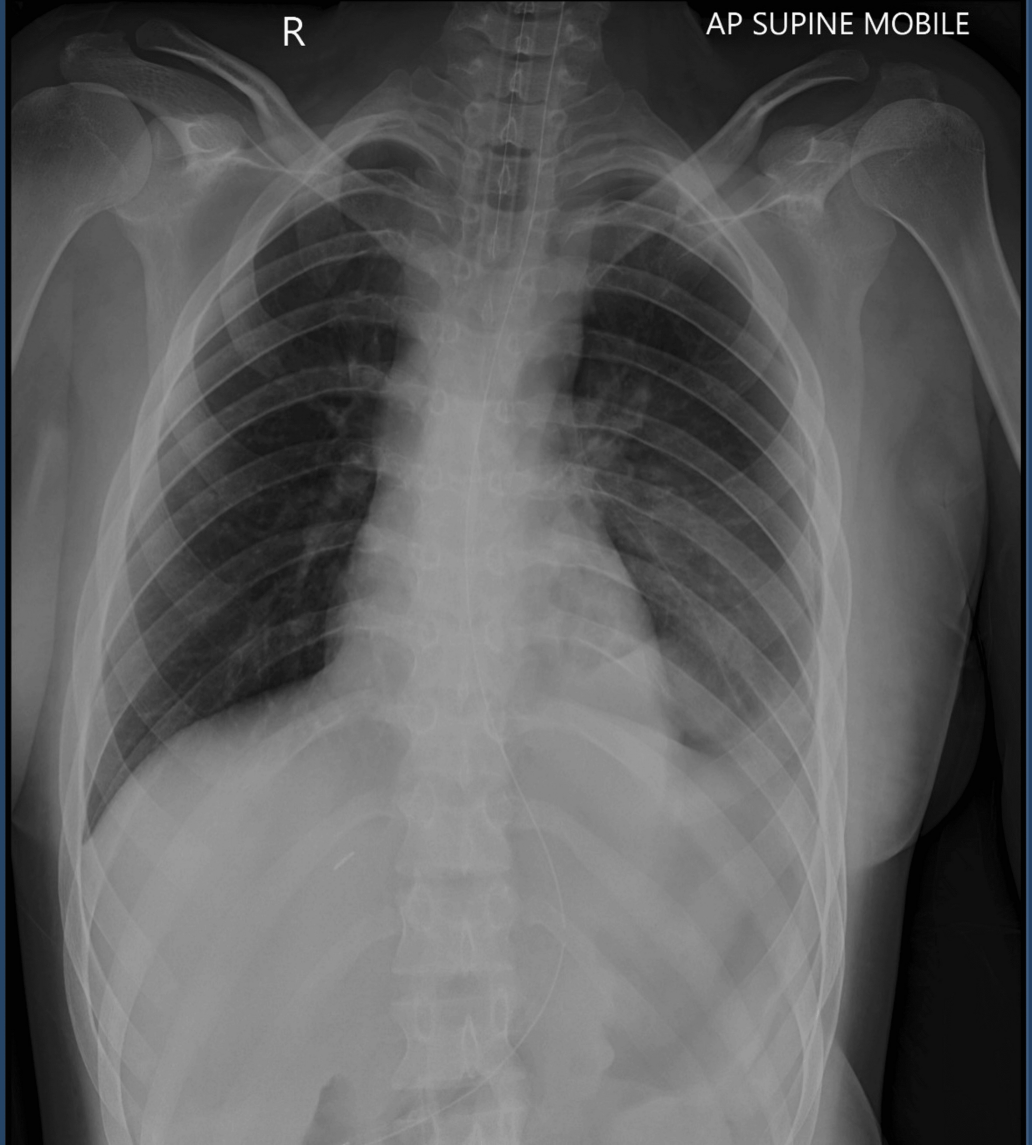
Postoperative chest radiograph (July 12, 2024) demonstrating satisfactory lung re-expansion with mild residual atelectasis and small postoperative pneumoperitoneum.

On July 12, 2024, postoperative chest radiography confirmed satisfactory lung re-expansion with mild residual atelectasis and a small pneumoperitoneum consistent with recent surgery (Figure 3). A nasogastric tube was left in place for free drainage, and broad-spectrum intravenous antibiotics were continued, along with intensive care-based analgesia.

The postoperative course was favorable, with inflammatory markers normalizing (CRP declined to 1.1 mg/L by August 2024), liver function stabilizing, and oral intake being resumed. At outpatient follow-up, the patient remained asymptomatic, with no evidence of recurrence and stable graft function under maintenance immunosuppression. A summary of the timeline of key clinical events, key findings, and management during hospitalization is presented in Table 2.

**Table 2:** Timeline summarizing the key clinical events, investigations, and management during hospitalization and follow-up. CRP, C-reactive protein; ESR, erythrocyte sedimentation rate; LFTs, liver function tests; CT, computed tomography; ECG, electrocardiogram

Date (2024)	Clinical course/symptoms	Key findings/investigations	Management and outcome
July 7	ED presentation with left arm pain, abdominal pain, and vomiting	Hb 11.1 g/dL, LFTs ↑, CRP 1.2 mg/L; CXR: bulging under L hemidiaphragm	Supportive care initiated; gastroenterology consult obtained; suspected gastritis.
July 8	Persistent abdominal pain and vomiting; ongoing left arm pain	Tacrolimus trough 6.9 ng/mL; abdominal ultrasound showed normal graft and trace pleural effusion; left arm ultrasound and X-ray were normal.	Orthopedic consultation: no acute pathology identified; tacrolimus dose increased; empiric intravenous ceftriaxone and vancomycin started.
July 9	Continued abdominal discomfort and left shoulder pain	Ultrasound of the left arm and abdomen showed normal solid organs and trace pleural effusion.	Supportive management and monitoring
July 10	Developed sudden nocturnal chest pain and shortness of breath	Chest X-ray showed persistent elevation of the left hemidiaphragm; ECG normal; liver enzymes trending downward.	Supplemental oxygen provided; observation and serial monitoring
July 11	Worsening left-sided chest pain and rising inflammatory markers	CRP 165 mg/L, ESR 51 mm/hour; CT of chest and abdomen showed a 2.5 cm defect in the left hemidiaphragm with herniation of the gastric fundus and associated pleural effusion.	Urgent surgical referral and underwent diaphragmatic hernia repair. Intraoperatively: viable stomach reduced; 400 mL of hemorrhagic pleural fluid aspirated; small adherent defect closed primarily with non-absorbable sutures.
July 12	Postoperative recovery	Nasogastric tube left in place for free drainage and broad-spectrum intravenous antibiotics continued, along with intensive care-based analgesia.	Postoperative chest X-ray showed lung re-expansion with mild atelectasis
July 13-August 12	Gradual postoperative recovery; resumed oral intake	CRP decreased to 1.1 mg/L; hemoglobin and liver enzymes normalized.	Transferred to her transplant center for continuation of care

## Discussion

Acquired DH following LT is a rare yet clinically important complication in pediatric recipients, with incidence rates in large cohorts reported between 0.8% and 2.9% [4,5,6]. The majority of cases involve the right hemidiaphragm, frequently linked to left lateral segment grafts and surgical stress at the diaphragm's bare area [3]. Left-sided DHs are distinctly less common, with only a few pediatric cases described worldwide [3]. Our case, therefore, adds to a small but growing body of literature documenting left-sided post-transplant DH in children. 

Several risk factors for post-transplant DH have been identified. The procedures include direct manipulation of the diaphragm during hepatectomy, the use of reduced or split grafts, and the implantation of large-for-size grafts [5]. Additional mechanisms, such as thermal or traction injury, devascularization, impaired wound healing due to immunosuppression, and sarcopenia or malnutrition, may also contribute [7]. Our patient demonstrated several of these factors, including long-term triple immunosuppression, prior graft complications, and young age at transplantation. The clinical presentation of DH may vary, potentially leading to delayed diagnosis. In pediatric patients, common symptoms include respiratory distress, abdominal pain, and vomiting [3]. Some, however, remain asymptomatic and are detected incidentally on imaging [7]. The patient initially presented with abdominal pain and vomiting, which were misattributed to gastritis, and subsequently developed chest pain and dyspnea. This case illustrates the diagnostic challenges and emphasizes the importance of considering DH in transplant recipients who experience recurrent or unexplained abdominal or respiratory symptoms.

Comparison with prior reports emphasizes several rare aspects of our case. Most published pediatric DHs after LT involve herniation of the small intestine [3,7,9], whereas our patient demonstrated herniation of the gastric fundus, complicated by pleural effusion and atelectasis. Gastric herniation is less common but clinically significant because of its higher risk of volvulus, ischemia, and perforation [6,7]. Moreover, most cases are diagnosed within the first post-transplant year, with median intervals of two to nine months [7]. In contrast, our patient was diagnosed more than two years post-transplant, underscoring that this complication may arise later in the clinical course.

Surgical repair remains the definitive management. Both laparotomy and minimally invasive techniques have been documented, with prompt reduction and secure closure of the defect being crucial to prevent recurrence [8,16]. In this case, thoracoscopic access was initially attempted but was converted to an open approach because of dense adhesions and challenges in reducing the herniated viscera. The gastric fundus and omentum were successfully reduced, the diaphragmatic defect was closed primarily, and the postoperative course was uneventful. This outcome is consistent with prior reports showing excellent prognosis when diagnosis is made early and surgical correction is prompt [4].

Geographically, most published pediatric DH cases after LT originate from high-volume centers in Turkey, Korea, China, and Brazil [5,7,13,16,17]. To the best of our knowledge, no reports of left-sided DH following pediatric LT have been reported from any Arab country within the Middle East, the GCC, or the UAE. This case was identified at the UAE’s only tertiary pediatric referral center, underscoring the relative rarity of this complication in the local setting and highlighting the importance of clinical vigilance in long-term follow-up.

In summary, this case illustrates the diagnostic and management challenges of post-transplant DH in children, while also contributing several unique elements to the literature: left-sided laterality, gastric herniation rather than small bowel, delayed presentation more than two years after transplantation, and being the first documented pediatric case from an Arab country in the Middle East. Clinicians caring for pediatric LT recipients should maintain a high index of suspicion for DH as a differential diagnosis in patients with unexplained chest or abdominal symptoms, regardless of time elapsed since transplantation. Early recognition, along with appropriate imaging and timely surgical repair, remains critical to optimizing outcomes.

## Conclusions

Post-transplant DH remains an uncommon but clinically important complication in pediatric LT, and its presentation may be subtle or significantly delayed. This case underscores the need for sustained clinical vigilance during long-term follow-up, particularly when atypical features such as left-sided defects or gastric involvement are encountered. The literature on left-sided DH in children is extremely limited, and reports from Arab Middle Eastern populations are nearly absent, highlighting a geographic gap in documented experience. As international reporting increases, there may be value in exploring early screening strategies for high-risk pediatric LT recipients. Important gaps persist regarding the precise risk profile, optimal surveillance approaches, and long-term outcomes. Future work should prioritize multicenter data collection, harmonized reporting, and targeted evaluation of high-risk subgroups to support the development of evidence-based recommendations for early detection and management. Increased reporting from underrepresented regions will be essential to strengthening the global evidence base. Improving clinician awareness may help reduce diagnostic delays and improve outcomes in this rare but serious complication.
